# vΔ50 Race Walking: High Energetic Cost, Rapid VO_2_max, and No Slow Component

**DOI:** 10.3390/jfmk11020174

**Published:** 2026-04-27

**Authors:** Laurence Mille-Hamard, Murielle Garcin, Stéphane Dufour, Véronique L. Billat

**Affiliations:** 1 Laboratoire Informatique, Bio-Informatique et Systèmes Complexes (IBISC), Université Paris-Saclay, 91034 Evry, France; veroniquelouisebillat@gmail.com; 2 Univ. Lille, CNRS, UMR 9193, SCALab, Sciences Cognitives et Sciences Affectives, 59000 Lille, France; 3 Faculty of Medicine, Translational Medicine Federation (FMTS), EA 3072, University of Strasbourg, 67000 Strasbourg, France; sdufour@unistra.fr; 4 Faculty of Sport Sciences, University of Strasbourg, 67000 Strasbourg, France

**Keywords:** race walking, oxygen uptake kinetics, VO2 slow component, energetic cost of locomotion, high-intensity interval training, severe-intensity exercise domain

## Abstract

**Background**: Race walking, an Olympic discipline, produces an increase in energy cost and a change in the recruitment pattern of muscle fibres compared with running, yet the cardiorespiratory responses of elite race walkers to severe-intensity exercise remain poorly characterised. **Objectives**: (i) To determine whether exhaustive exercise performed at vΔ50 elicits VO_2_max in young elite race walkers, and (ii) to compare the temporal and metabolic profiles of this effort with those of similarly trained runners. **Methods**: Fourteen elite junior athletes (seven race walkers and seven runners) completed an incremental test to determine velocity at the lactate threshold (vLT), vVO2max, and VO2max, followed by a constant-velocity trial at individual vΔ50 performed to voluntary exhaustion on a 400 m track. Breath-by-breath VO2, heart rate, capillary blood lactate concentration, and time to exhaustion, time limit (Tlim) were measured. **Results**: At vΔ50 (≈94% vVO2max), the race walkers reached VO2max, with no detectable VO2 slow component (SC) in six of seven participants. In contrast, runners exhibited a significant SC (8 ± 3% of total VO2). The energy cost (EC) was 16% higher in race walking than in running (*p* < 0.01). **Conclusions**: In elite junior race walkers, it seems that vΔ50 reliably elicits VO2max primarily due to a high baseline oxygen cost rather than a progressive VO2 SC, contrasting with the kinetic response observed in running. These discipline-specific responses suggest that interval training in race walking should be prescribed using walking-specific thresholds. This study is preliminary, given the small sample size; further studies with larger cohorts are warranted.

## 1. Introduction

Race walking is the only Olympic discipline in which athletes are required to sustain long-distance locomotion at competitive speeds while maintaining continuous ground contact. According to World Athletics Rule 54, visible loss of contact with the ground is prohibited, and the stance leg must remain straight from initial contact until the body passes directly over it, thereby eliminating the aerial—or “flight”—phase that characterises running [1]. These technical constraints impose distinct biomechanical demands with potential physiological consequences that remain incompletely understood. The absence of an aerial phase induces two primary biomechanical effects. First, vertical displacement of the centre of mass is markedly reduced, which lowers peak ground reaction forces but concurrently limits the storage and subsequent release of elastic strain energy within the muscle–tendon units during stance [2,3]. Second, the step frequency must increase substantially to attain race-walking velocities that frequently exceed 4 m·s^−1^ (≈14–15 km·h^−1^) [2,3]. Together, these adaptations elevate the net metabolic cost of transport at speeds above approximately 3 m·s^−1^, rendering race walking one of the most energetically demanding modes of terrestrial locomotion [2,3,4]. Paradoxically, elite race walkers demonstrate maximal oxygen uptake (VO_2_max) values (≈70–80 mL·kg^−1^·min^−1^) comparable to those observed in similarly ranked distance runners [5,6]. Together with a high lactate threshold and refined technical proficiency, this level of aerobic power underpins performance in the Olympic 20 km and 35 km race-walking events [7,8]. Athletes typically develop this capacity through high training volumes of event-specific walking, complemented by high-intensity interval training (HIIT) protocols originally derived from running, such as repeated efforts lasting 30 s to 4 min at or above the velocity eliciting VO_2_max (vVO_2_max), interspersed with short recovery intervals [9,10].

Although more than five decades of research have delineated well-defined physiological training zones for running, evidence-based prescriptions for HIIT in race walking remain limited. Current practice frequently applies velocity or heart rate targets validated in running without verification of their suitability within the mechanically distinct constraints of race walking. Such extrapolation may be inappropriate, as differences in muscle recruitment patterns, fascicle behaviour, and elastic energy storage and return are likely to modify cardiorespiratory responses at a given relative intensity. Accurate classification of training intensity becomes particularly important within the heavy-to-severe intensity domain [11,12], which is bounded by the second ventilatory threshold and vVO_2_max. In runners, the midpoint between vLT and vVO_2_max—termed vΔ50—has been shown to provide a practical reference for both constant-speed time-to-exhaustion testing and the prescription of interval-training workloads [13,14]. Whether this running-derived benchmark can be directly transferred to race walking remains uncertain, as the validity of physiological intensity anchors is contingent on the locomotor modality in which they were established.

An additional layer of complexity arises from the kinetics of oxygen uptake (VO_2_). VO_2_ is not solely determined by the external work rate but also evolves over time, particularly at exercise intensities above the second ventilatory threshold, where the so-called slow component (SC) induces a progressive increase in VO_2_ [11,15]. This phenomenon elevates the effective oxygen cost of exercise and reduces the time limt (Tlim). In running, Tlim measured at vVO_2_max or at vΔ50 is commonly used to individualise the duration of HIIT work bouts [13]. However, no study has yet characterised VO_2_ kinetics or Tlim in race walkers exercising at vΔ50. Contraction patterns and muscle fibre recruitment strategies have been proposed as key determinants of both the presence and the magnitude of the VO_2_ SC [16,17]. Accordingly, there is a clear need to quantify the physiological responses of race walkers exercising at vΔ50 and to compare these responses with those of runners matched for age, sex, and VO_2_max. Such knowledge would inform the development of walking-specific HIIT protocols, refine the definition of training intensity zones, and ultimately contribute to performance optimisation in this Olympic discipline.

The aims of the present study were, therefore, (i) to determine whether exhaustive exercise performed at vΔ50 elicits VO_2_max in young elite race walkers and (ii) to compare the temporal and metabolic profiles of this exercise with those observed in equivalently trained runners. It was further hypothesised that, despite exercising at the same relative intensity, race walkers would reach VO_2_max more rapidly and achieve volitional exhaustion later than runners, due to a higher baseline locomotor cost and a less pronounced VO_2_ SC.

## 2. Materials and Methods

### 2.1. Study Design

This exploratory cross-sectional study used a between-group observational design in which two groups of athletes—race walkers and runners—were tested under comparable experimental conditions ([18]). Each group performed all trials using their respective locomotion mode. The between-group design was selected to avoid confusion between the effects of locomotion mode and those of occasional exposure to an unusual gait. Each participant completed two exercise tests separated by 48 h, and they were instructed not to engage in intensive training sessions between the two tests: (1) an incremental test to determine VO_2_max, vVO_2_max, and vLT, and (2) a constant-velocity exhaustion trial at vΔ50. All tests were conducted on the same outdoor 400m synthetic running track, under similar environmental conditions (ambient temperature: 14–19 °C; wind speed < 3 m·s^−1^). Participants were instructed to arrive rested, well-hydrated, and at least 3 h post-prandial.

### 2.2. Participants

Fourteen volunteer junior athletes (height: 166.1 ± 7.5 cm; body mass: 54.8 ± 7.5 kg) were recruited for this study (Table 1). They trained between three and five times per week, for approximately 45 min per session, and had practised their respective activity for at least 8 years. All these athletes were members of the regional/national team.

Participants were classified as runners (R; 4 females and 3 males) or race walkers (W; 4 females and 3 males). The two groups were matched for VO_2_max (59.2 ± 7.9 mL·kg^−1^·min^−1^ for W vs. 58.9 ± 8.5 mL·kg^−1^·min^−1^ for R; *p* = 0.813), age (19.7 ± 3.8 years for W vs. 18.4 ± 2.4 years for R; *p* = 0.434), and sex.

All participants provided written informed consent prior to participation. This study was conducted in accordance with the Declaration of Helsinki. Ethical approval was granted by the Institutional Review Board of Université de Lille (approval reference: CP 00/10; date of approval: 2000).

### 2.3. Instrumentation and Measurements

Respiratory gas exchange was measured breath-by-breath using a portable metabolic analyser (K4b2, Cosmed, Rome, Italy), calibrated before each test according to the manufacturer’s instructions. The turbine flow meter was calibrated using a 3 L syringe (Quinton Instruments^®^, Seattle, WA, USA). Published field reliability data for this system report a coefficient of variation for VO_2_ of approximately 3% during submaximal exercise [see the manufacturer’s validation documentation]; field reliability under outdoor conditions may slightly exceed laboratory estimates. Heart rate (HR) was recorded continuously via a chest strap monitor. Blood lactate concentration ([La]) was determined from fingertip capillary samples using an enzymatic spectrophotometric assay (Dr Lange^®^ GmbH, Berlin, Germany), verified before each measurement using lactate standard solutions.

Speed was controlled using a pacing cyclist who received audio cues at intervals corresponding to the time required to cover 25 m at the target velocity. Visual markers were placed every 25 m along the inside of the first lane. Lap split times were recorded by an independent timekeeper; the mean deviation from the target speed was below 0.2 km·h^−1^ across all participants.

### 2.4. Incremental Test

The participants performed a discontinuous incremental test with 3 min stages separated by 30 s rest intervals for capillary blood sampling. This stage duration and rest design is consistent with previous race-walking physiology research [19,20] and enables reliable lactate profiling under field conditions; however, it is acknowledged that brief rest intervals may slightly alter lactate kinetics compared with continuous protocols. The starting velocity was set approximately 6 km·h^−1^ below the estimated vVO_2_max (based on the best 3000 m performance), and the velocity was increased by 1 km·h^−1^ at the end of each stage, ensuring exhaustion occurred within approximately 20 min. Additional blood samples were collected before the test, at the end of the test, and 3 min post-exercise. The participants received standardised verbal encouragement throughout.

VO_2_max was defined as the highest 15 s-averaged VO_2_ value attained during the incremental test. vVO_2_max was defined as the lowest velocity at which VO_2_max was achieved. The velocity at the lactate threshold (vLT) was identified as the velocity corresponding to an increase of 1 mmol·L^−1^ in blood lactate concentration when [La] values ranged between 3.5 and 5 mmol·L^−1^. This operational criterion was selected for its practical applicability under field conditions and its alignment with prior research [21], whilst acknowledging that it differed from the more commonly used fixed threshold (e.g., 4 mmol·L^−1^) or individual anaerobic threshold approaches. The measurement variability associated with capillary lactate sampling (±0.2 mmol·L^−1^) may translate into an uncertainty of approximately 0.1–0.2 km·h^−1^ in vLT, which propagates to vΔ50 estimation.

### 2.5. Constant-Velocity Trial at vΔ50

Two days after the incremental test, participants completed a high-intensity constant-load test to exhaustion. The exercise intensity was set at vΔ50, defined as the arithmetic midpoint between vVO_2_max and vLT. This parameter has been validated as a practical intensity anchor for severe-domain exercise in runners [13,14]; its use in race walking is exploratory, given that no prior study has confirmed the physiological equivalence of vΔ50 across locomotor modalities. The present study was designed, in part, to provide this validation.

After a 15 min warm-up at 60% vVO_2_max and a 5 min passive rest period, participants were instructed to maintain the prescribed velocity until volitional exhaustion. Exhaustion was operationally defined as volitional termination signalled by the participant or failure to maintain the prescribed speed (when the athlete was more than 5 m behind the cyclist for at least 100 m despite standardised verbal encouragement). Tlim was recorded as the total duration of the constant-velocity phase. A single trial was conducted per participant due to the logistical constraints of recruiting elite junior athletes during their competitive season; the absence of repeated-measure reliability data should be considered when interpreting individual results.

### 2.6. VO_2_ Kinetics Analysis

Breath-by-breath VO_2_ data were screened for artefacts prior to analysis. Breaths deviating by more than 3 SD from the local mean in a ±10-breath window were removed (<2% of breaths per trial). The data were then averaged over 5 s intervals and smoothed with a three-point moving average filter to reduce noise whilst preserving the underlying physiological signal (Data Management Software, Cosmed, Italy). VO_2_ kinetics were subsequently modelled using the SigmaPlot software version 15 (SPSS Inc., Chicago, IL, USA).

Two models were fitted to the VO_2_ time-series data [22,23]:

Equation (1) (mono-exponential):

VO_2_ (t) = A_0_ + A × (1 − e ^[−(t−TD)/τ]^) × u, (1)

where u = 0 when t < TD, u = 1 when t ≥ TD, A_0_is the baseline VO_2_, A is the asymptotic amplitude, τ is the time constant, and TD is the time delay. The early cardiodynamic phase was not modelled, as it does not influence primary component amplitude or the subsequent time course of VO_2_ kinetics [24].

Equation (2) (bi-exponential):

VO_2_ (t) = A_0_ + A_1_ × (1 − e^[−(t−TD1)/τ1]^) × u_1_ + A_2_ × (1 − e^[−(t−TD2)/τ2]^) × u_2_, (2)

where index 1 describes the primary component (A_1_, τ_1_, and TD_1_), and index 2 describes the SC (A_2_, τ_2_, and TD_2_).

The amplitude of the primary component at the onset of the SC was

A’_1_ = A_1_ × (1 − e ^[−(TD2−TD1)/τ1]^), (3)

and the SC amplitude was

A’_2_ = A_2_ × (1 − e ^[−(te−TD2)/τ2]^), (4)

where te denotes the time at the end of exercise.

Parameters were estimated using iterative non-linear least-squares minimisation. Model selection was based on Fisher’s F-test. The bi-exponential model was preferred when the F-ratio was statistically significant (*p* < 0.05), indicating a meaningful improvement in fit over the mono-exponential model.

### 2.7. Net Aerobic Energy Cost of Locomotion

The aerobic energy cost (EC) of locomotion was calculated at two speeds: (i) at a sub-threshold speed during the incremental test (EC_subT_), and (ii) at vΔ50 during the constant-velocity trial (EC_vΔ50_). The formula used was

EC = (maxVO_2_ − VO_2_rest)/v, (5)

where maxVO_2_ is the maximal VO_2_ (mL·kg^−1^·min^−1^) attained either during the constant-velocity trial or during the submaximal stage of the incremental test; VO_2_rest is the resting VO_2_ assumed to be 5 mL·kg^−1^·min^−1^ (corresponding to the y-intercept of the VO_2_–speed regression line [25]); and v is the corresponding velocity in m·min^−1^. EC is expressed in mL·kg^−1^·m^−1^. The use of a standardised resting VO_2_ rather than individual measurements is consistent with prior EC studies in race walking [20,26] and avoids error associated with measuring resting VO_2_ in a field setting; however, this assumption may introduce a small systematic error in participants whose true resting metabolic rate deviates from the population mean.

### 2.8. Statistical Analysis

Non-parametric statistics were used throughout, given the small sample size (n = 7 per group), which precluded adequate verification of normality assumptions. The data are presented as medians, interquartile ranges (IQRs) and 95% confidence intervals (CIs). Between-group comparisons were performed using the Mann–Whitney U test; within-group comparisons used the Wilcoxon signed-rank test.

Cohen’s d effect sizes (ESs) were calculated to quantify the magnitude of between-group and within-group differences, interpreted as small (0.2 ≤ d < 0.5), moderate (0.5 ≤ d < 0.8), and large (d ≥ 0.8) [27]. It should be noted that Cohen’s d estimates are unstable in small samples, and confidence intervals around effect size estimates are wide; effect sizes are, therefore, reported descriptively and should not be over-interpreted. Fisher’s exact test was used for model selection (mono- vs. bi-exponential), and a chi-square test compared model fit proportions between groups. Statistical significance was set at *p* < 0.05. All analyses were performed using the SigmaPlot software version 15 (SPSS Inc., Chicago, IL, USA).

## 3. Results

The mean values of the physiological variables obtained during the incremental exercise tests are presented in Table 2. These data confirmed the comparability of the two groups at baseline. The velocity associated with VO_2_max differed significantly between groups (*p* < 0.001 and large effect size). For the remaining variables, no statistically significant differences were observed under the conditions of this study; corresponding effect sizes were negligible for VO_2_max and small for heart rate and blood lactate concentration (Table 2). The effect sizes throughout should be interpreted cautiously, given the small sample size (n = 7 per group), which limits their precision.

The characteristics of the constant-velocity exercise and the associated physiological responses are presented in Table 3. The absolute velocity at vΔ50 differed significantly between groups, with a large effect size (ES = 1.5), reflecting the substantially lower race-walking speed relative to running. However, when expressed as a percentage of vVO_2_max, no between-group difference was observed (*p* = 0.266; ES < 0.2), suggesting equivalent relative exercise intensity. No statistically significant differences were found for HR_max_, [La]_max_, or Tlim; the corresponding effect sizes ranged from negligible (Tlim) to moderate ([La]_max_). However, this absence of statistically significant differences should not be interpreted as evidence of equivalence, due to the small sample size.

In all participants, similar maximal values of VO_2_ and heart rate were achieved during the incremental and constant-velocity trials (*p* = 0.33 and *p* = 0.31, respectively), with ES < 0.2 for VO_2_ and ES = 0.2 for heart rate, under the present experimental conditions. The EC of race walking was significantly higher than that of running at high intensity (0.274 ± 25 vs. 0.198 ± 16 mL·kg^−1^·m^−1^; *p* < 0.001; ES = 1.6), and increased significantly (+16%) from moderate to high speeds (*p* = 0.014; ES = 1.7; Table 4). No significant intensity-related change in EC was observed in runners (*p* = 0.17; ES < 0.2).

**Figure 1:**
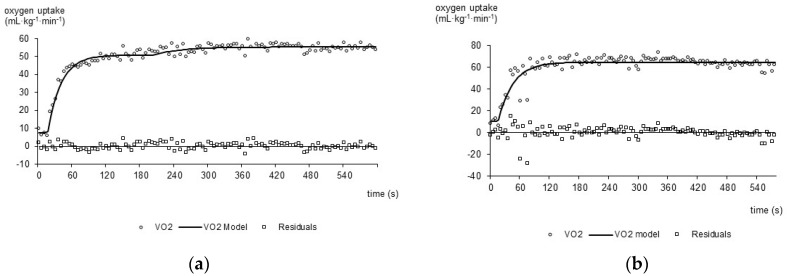
Best-fit oxygen uptake curves during the constant-velocity trial, with residual plots (lower panels), for a representative runner fitted with a bi-exponential model (**a**) and a representative walker fitted with a mono-exponential model (**b**). Residuals distributed randomly around zero indicate adequate model fit.

In the running group, the VO_2_ response was best described by the bi-exponential model (Figure 1a) in five of seven participants, indicating the presence of a VO_2_ SC. In the remaining two runners, the mono-exponential model provided the better fit (Figure 1b). In the race-walking group, only one of seven participants was best described by the bi-exponential model; the remaining six were best fitted by a mono-exponential function. The distribution of model fits differed significantly between groups (chi-square test, *p* = 0.03). Individual VO_2_ kinetic parameters are presented in Table 5, which also highlights the substantial inter-individual variability in base line oxygen uptake, amplitude, and time-constant values within both groups, a feature that warrants attention in future studies with larger samples.

No statistically significant between-group differences were observed in the parameters of the primary VO_2_ component within the present experimental framework (Table 5). The overall VO_2_ amplitude (A’_1_ or A’_1_ + A’_2_) was similar in both groups (*p* = 0.80; ES = 0.3).

In the five runners exhibiting a VO_2_ SC, the SC amplitude was not significantly correlated with end-exercise blood lactate (*p* = 0.060) or Tlim (*p* = 0.661). The 16% increase in the EC of race walking from the sub-threshold to vΔ50 intensity was not attributable to a VO_2_ SC, given that a SC was observed in only one of seven walkers.

## 4. Discussion

### 4.1. Principal Findings

The present study indicates that, in elite young race walkers, an exhaustive constant-velocity bout at vΔ50—the midpoint between vLT and vVO_2_max—seems sufficient to elicit VO_2_max, consistent with what has previously been shown in running [28,29]. Importantly, these findings suggest—with the caveat of a small sample—that vΔ50 may represent a physiologically grounded anchor for HIIT prescription in race walking, a discipline in which intensity zones have historically relied on extrapolation from running. The matched-group design further demonstrates that, although walkers and runners exercised at comparable relative intensities (vΔ50 ≈ 94% vVO_2_max), the time course of VO_2_ and the energetic cost (EC) of locomotion diverged markedly between gaits.

### 4.2. Context Within the Literature

Despite its Olympic status, competitive race walking has received limited scientific attention compared with marathon or middle-distance running. The existing literature is largely dominated by biomechanical investigations emphasising the technical demands of maintaining legal form at jogging-comparable velocities [30]. The present study contributes novel physiological insight by providing, for the first time, breath-by-breath VO_2_ kinetics during severe-intensity race walking under field conditions. The findings confirm earlier reports of a marked increase in EC above ≈3 m·s^−1^ [19,20], and extend these observations by disentangling the baseline EC from any contribution of a VO_2_ SC.

### 4.3. The VO_2_ Slow Component

Exercise intensity domains are commonly classified as moderate (no SC), heavy (SC present but VO_2_max not attained), and severe (SC leading to VO_2_max) [31,32]. In the present study, five of seven runners showed a measurable SC (≈8% of total VO_2_), consistent with prior observations during running at 90–95% vVO_2_max [12,33]. In contrast, six of seven race walkers showed no detectable SC despite reaching VO_2_max, a pattern that, if replicated, would suggest different mechanisms underlie the attainment of VO_2_max in the two locomotion modes.

In running, the SC has been attributed to progressive recruitment of type II muscle fibres, an increased ATP cost of force production, and changes in elastic energy utilisation [33,34,35,36]. Race walking is characterised by lower vertical ground reaction forces and likely different fascicle length dynamics [2,3], features that may limit high-threshold motor unit recruitment and thereby attenuate SC development. It should be emphasised, however, that no direct measurements of muscle activation (EMG) or fibre-type recruitment were obtained in the present study; mechanistic interpretations should, therefore, be regarded as plausible, hypothesis-generating explanations rather than established findings. An alternative interpretation is that the observation window (≈11 min median Tlim for walkers) may have been insufficient to capture a late-onset SC, which, in some studies, emerges only after 5–10 min of severe-intensity exercise. Furthermore, individual differences in muscle metabolic efficiency, inter-muscular coordination, and thermal dissipation under field conditions represent plausible alternative contributors to the between-group difference in SC prevalence. Future studies combining direct neuromuscular measurement with longer exercise bouts are needed to distinguish among these possibilities.

The elevated EC of race walking cannot be attributed to the development of a VO_2_ SC. Rather, it could reflect the mechanical constraints imposed by Rule 54: the continuous ground contact requirement precludes elastic energy storage and reutilisation, obliging greater reliance on concentric muscle work, while step frequencies exceeding 200 strides·min^−1^ substantially increase the metabolic cost of calcium handling and cross-bridge cycling [37,38]. These constraints seem to generate a high baseline oxygen demand that appears sufficient to drive VO_2_ to its maximum without a time-dependent drift. The present findings align with cross-modality comparisons in cycling, swimming, and running, suggesting that SC magnitude increases with active muscle mass and/or contraction velocity [12,39,40], and with the inverse relationship between type I fibre proportion and SC amplitude [31,41,42], though, again, these parallels are correlational rather than mechanistic.

### 4.4. Energetic Cost and Biomechanical Determinants

The 16% increase in EC from sub-threshold to vΔ50 in walkers mirrors the 13–35% increments reported by Chwała et al. across technical, threshold, and competition paces [19], indicating that small speed increments above the lactate threshold impose a disproportionate metabolic penalty in race walking. Fatigue-related biomechanical alterations (increased ground contact time and reduced leg stiffness) exacerbate EC and SC in running and cycling [43,44]; in race walking, equivalent fatigue markers remain understudied. Although participants covered only ≈3 km in the present study, technique degradation over championship distances (10–35 km) has been documented [45,46] and may further elevate EC, providing a physiological rationale for conservative early pacing strategies.

### 4.5. Strengths of This Study

Several methodological strengths should be acknowledged. First, the matched-group design—with groups equated for VO_2_max, age, and sex composition—allowed a meaningful comparison of locomotor modalities at equivalent relative intensities. Second, the use of a 400 m outdoor track enhanced ecological validity, ensuring that physiological responses reflected genuine race-walking conditions rather than laboratory treadmill artefacts. Third, breath-by-breath gas analysis enabled high-resolution kinetic modelling of VO_2_ responses. Fourth, the recruitment of nationally competitive junior athletes ensured that findings are relevant to elite-level practice.

### 4.6. Training and Performance Implications

The present findings, whilst preliminary, offer useful guidance for practitioners working with elite race walkers. HIIT intervals prescribed at individual vΔ50 appeared to elicit VO_2_max within approximately 2–3 min in the present cohort, suggesting that traditional 2–3-min work intervals with equal or slightly shorter recovery periods may provide a potent oxidative stimulus. Because VO_2_max was achieved without reliance on a progressive SC, the aerobic stimulus accumulates rapidly; excessively prolonged intervals may, therefore, add mechanical stress without proportional physiological benefit. Importantly, the transposition of running-derived heart rate or speed thresholds to race walking should be avoided. Despite similar relative intensities, identical absolute velocities imposed markedly different metabolic demands across gaits. These practical recommendations should be viewed as conditional and provisional, pending replication in larger and more diverse cohorts.

### 4.7. Limitations

Several limitations temper the generalisability of these findings and warrant explicit acknowledgement.

First, the sample size was small (n = 7 per group), reflecting the limited availability of elite junior race walkers. Although the effect sizes for the primary outcomes were large, some analyses were likely underpowered and susceptible to type II error; effect size estimates should be treated with caution, and this study must be considered as preliminary.

Second, the between-group design cannot cleanly separate the effects of locomotor modality from those of long-term, sport-specific physiological adaptation. Differences in VO_2_ kinetics between walkers and runners may reflect training history, fibre-type composition, or other discipline-specific adaptations as much as the biomechanical constraints of the gait per se.

Third, the cohort included both female (n = 4) and male (n = 3) participants per group. The sample was too small to conduct adequately powered sex-stratified analyses; potential sex differences in VO_2_ kinetics and EC—which have been reported in running—cannot be excluded and should be examined in future research with a larger sample size.

Fourth, muscle–tendon dynamics and motor unit recruitment were inferred rather than directly measured. Future studies integrating surface EMG, ultrasound imaging, and ^31^P-MRS would allow direct examination of the neuromuscular mechanisms underlying the absence of an SC in race walking.

Fifth, the outdoor track setting—whilst enhancing ecological validity—introduced uncontrolled environmental variability (wind and temperature) and minor pacing fluctuations that may have influenced breath-by-breath VO_2_ kinetics.

Sixth, a single exhaustion trial was conducted per participant; without repeated trials, the within-subject reliability of kinetic parameters cannot be confirmed. However, at vΔ50, the intra-individual variability was less than 10% [47].

Finally, the results pertain to young, nationally competitive athletes; senior or masters race walkers may exhibit different kinetics due to long-term adaptations or age-related changes in tendon stiffness.

### 4.8. Perspectives and Future Research

The mechanistic basis of the absent SC in race walking warrants deeper investigation. Longitudinal intervention studies could determine whether vΔ50-based HIIT induces adaptations in muscle phenotype, tendon elastic properties, or neuromuscular coordination. Wearable metabolic analysers combined with inertial measurement units would enable in-competition monitoring of VO_2_ kinetics over 10–35 km, testing whether short-duration laboratory findings extrapolate to competitive race distances. Beyond elite sport, reducing the SC in clinical or ageing populations could improve functional mobility by lowering the metabolic cost of locomotion.

## 5. Conclusions

These findings suggest that exhaustive race walking at vΔ50 can elicit VO_2_max via a high baseline metabolic cost rather than a progressive VO_2_ SC—a pathway that appears to distinguish race walking from running at comparable fractional intensities. Pending replication in larger cohorts, vΔ50 derived from walking-specific thresholds may represent a useful anchor for HIIT prescription in race walking. The unique interplay between technical rules, biomechanical constraints, and VO_2_ kinetics in race walking underscores the need for discipline-specific approaches to training intensity prescription and points to a rich agenda for future physiological and mechanistic research in this understudied Olympic discipline.

## Figures and Tables

**Table 1:** Anthropometric characteristics of the group of walkers (W) and the group of runners (R). IQR: interquartile range; CI: confidence interval.

	Age (years)		Height (cm)		Weight (kg)	
Sport	W	R	W	R	W	R
Median	19.7	18.4	163.3	168.9	56.6	53.0
IQR	3.8	2.4	7.4	7.5	5.9	7.5
95% CI	16–20	16–22	163–175	156–166	46–58	52–60

**Table 2:** Characteristics of the incremental exercise test in the walker (W) and runner (R) groups. vVO2max: velocity at maximal oxygen uptake; VO2max: maximal oxygen uptake; HRmax: maximal heart rate; [La]max: maximal blood lactate concentration; IQR; interquartile range; CI: confidence interval; ES: effect size.

	vVO_2max_ (km·h^−1^)		VO_2max_ (mL·kg^−1^·min^−1^)		HR_max_ (bt·min^−1^)		[La]_max_ (mM)	
Sport	W	R	W	R	W	R	W	R
Median	13	17	59.0	59.1	199	197	10.2	11.6
IQR	1.5	3.0	6.2	8.8	6.5	3.5	1.2	3.3
95% IC	13.0–13.0	17.0–17.0	58.8–59.2	58.9–59.3	199.4–199.7	196.9–197.1	10.2–10.2	11.5–11.7
ES	1.6		<0.2		0.2		0.3	
*p*	<0.001		0.81		0.33		0.52	

**Table 3:** Characteristics of the constant-velocity exhaustive exercise trial in walkers (W) and runners (R). vΔ50: midpoint velocity between vVO_2_max and vLT; HR_max_: maximal heart rate; [La]_max_: maximal blood lactate; Tlim: time to exhaustion; IQR; interquartile range; CI: confidence interval; ES: effect size.

	vΔ50 (km·h^−1^)		v Δ50 (% vVO_2max_)		Max HR (bt·min^−1^)		Max La (mM)		Tlim (sec)	
	W	R	W	R	W	R	W	R	W	R
**Median**	12	15.5	93.0	92.3	198	193	9.5	13	685	555
**IQR**	1.0	3.0	2.0	4.0	6.0	4.5	3.7	3.2	280	205
**95% CI**	12.0–12.0	15.4–15.6	92.9–93.1	92.2–92.4	193.3–202.7	192.8–193.2	9.4–9.6	12.9–13.1	668–701	542–567
**ES**	1.5		<0.2		0.2		0.5		<0.2	
***p***	0.001		0.266		0.700		0.400		0.958	

**Table 4:** Energy cost (EC) calculated at sub-threshold intensity (EC_subT_) and at vΔ50 (EC_vΔ50_), together with the maximal VO_2_ attained during the constant-velocity trial in walkers (W) and runners (R). IQR: interquartile range; CI: confidence interval; ES: effect size.

	max VO_2_ (mL·kg^−1^·min^−1^)		EC at vΔ50 (mL·kg^−1^·m^−1^)		EC subT (mL·kg^−1^·m^−1^)	
	W	R	W	R	W	R
**Median**	57.2	57.0	0.271	0.198	0.238	0.192
**IQR**	4.3	2.7	0.026	0.020		
**95% CI**	57.1–57.3	56.8–57.2	0.270–0.272	0.202–0.202	0.237–0.239	0.191–0.193
**ES**	0.3		1.6		1.2	
***p***	0.354		<0.001		0.18	

**Table 5:** Individual parameters from exponential curve fitting of VO_2_ responses for walkers (W) and runners (R) during the constant-velocity trial. TD_1_ and TD_2_: time delay for each component; A’_1_ and A’_2_: amplitude of each component; τ_1_ and τ_2_: time constant of each component; VO_2_b: baseline oxygen uptake. IQR: Dashes (–) indicate that the bi-exponential model did not converge (i.e., no SC was identified) for that participant; IQR: interquartile range; CI: confidence interval; ES: effect size.

	TD_1_ (s)		VO_2_b (mL·min^−1^)		A’_1_ (mL·min^−1^)		τ_1_ (s)		TD_2_ (s)		A’_2_ (mL·min^−1^)		τ_2_ (s)		A’_1_ + A’_2_ (mL·min^−1^)	
	W	R	W	R	W	R	W	R	W	R	W	R	W	R	W	R
1	18.0	9.0	676	285	1920	2022	41.7	26.5	-	276	-	139	-	141	1920	2161
2	14.3	17.7	280	468	2292	1853	11.0	26.3	-	200	-	200	-	172	2292	2053
3	20.4	14.6	448	367	2057	2070	30.9	23.5	-	200	-	217	-	50	2057	2287
4	13.0	28.0	276	528	2385	2201	22.1	18.0	-	-	-	-	-	-	2385	2201
5	20.8	16.5	420	772	2067	2800	41.2	20.7	-	-	-	-	-	-	2067	2800
6	14.4	10.0	652	754	3299	2868	29.7	41.0	-	200	-	366	-	95	3299	3234
7	5.0	14.1	472	322	2823	1932	29.7	16.4	200	282	192	50	50	100	3015	1982
Median	14.4	14.6	448	468	2292	2070	29.7	23.5	-	200	-	200	-	100	2292	2201
IQR	5.5	5.1	212	296	542	524	10.2	7.1		76		78		46	638	437
95%CI	10.4–18.4	9.9–19.3	330–565	321–615	1763–2377	22–38	-	17–30	-	168–232	-	114–286	-	65–135	1904–2680	1862–2540
*p*	0.90		0.62		0.53		0.21		-		-		-		0.80	
ES	0.1		0.4		<0.2		0.4		-		-		-		0.3	

## Data Availability

The original contributions presented in this study are included in the article. Further inquiries can be directed to the corresponding author.

## Abbreviations

The following abbreviations are used in this manuscript:

SC	Slow component
VO_2_	Oxygen uptake
VO_2_max	Maximal oxygen uptake
vVO_2_max	Velocity at VO_2_max
vLT	Velocity at lactate threshold
vΔ50	Velocity midway between vVO_2_max and vLT
HIIT	High-intensity interval training
Tlim	Time to exhaustion
R	Runners group
W	Walkers group
EC	Energy cost
ES	Effect size
HR	Heart rate
CI	Confidence interval
